# *In vivo* vascularization of MSC-loaded porous hydroxyapatite constructs coated with VEGF-functionalized collagen/heparin multilayers

**DOI:** 10.1038/srep19871

**Published:** 2016-01-22

**Authors:** Kai Jin, Bo Li, Lixia Lou, Yufeng Xu, Xin Ye, Ke Yao, Juan Ye, Changyou Gao

**Affiliations:** 1 Department of Ophthalmology, the Second Affiliated Hospital of Zhejiang University, College of Medicine, Hangzhou 310009, China; 2 MOE Key Laboratory of Macromolecular Synthesis and Functionalization, Department of Polymer Science and Engineering, Zhejiang University, Hangzhou 310027, China

## Abstract

Rapid and adequate vascularization is vital to the long-term success of porous orbital enucleation implants. In this study, porous hydroxyapatite (HA) scaffolds coated with vascular endothelial growth factor (VEGF)-functionalized collagen (COL)/heparin (HEP) multilayers (porosity 75%, pore size 316.8 ± 77.1 μm, VEGF dose 3.39 ng/mm^3^) were fabricated to enhance vascularization by inducing the differentiation of mesenchymal stem cells (MSCs) to endothelial cells. The *in vitro* immunofluorescence staining, quantitative real-time polymerase chain reaction (qRT-PCR), and western blotting results demonstrated that the expression of the endothelial differentiation markers CD31, Flk-1, and von Willebrand factor (vWF) was significantly increased in the HA/(COL/HEP)_5_/VEGF/MSCs group compared with the HA/VEGF/MSCs group. Moreover, the HA/(COL/HEP)_5_ scaffolds showed a better entrapment of the MSCs and accelerated cell proliferation. The *in vivo* assays showed that the number of newly formed vessels within the constructs after 28 d was significantly higher in the HA/(COL/HEP)_5_/VEGF/MSCs group (51.9 ± 6.3/mm^2^) than in the HA (26.7 ± 2.3/mm^2^) and HA/VEGF/MSCs (38.2 ± 2.4/mm^2^) groups. The qRT-PCR and western blotting results demonstrated that the HA/(COL/HEP)_5_/VEGF/MSCs group also had the highest expression of CD31, Flk-1, and vWF at both the mRNA and protein levels.

The removal of an eye may be necessary in cases of severe ocular trauma, intraocular malignancy, blind painful eye, prevention of sympathetic ophthalmia, and cosmesis. Currently, most enucleated patients can confidently return to their daily activities by inserting an orbital implant into the socket after enucleation or evisceration or by wearing an ocular prosthesis. However, sufficient vascularization of the thick implants, such as coralline porous hydroxyapatite (HA) spheres, remains a key challenge^1^. Although many successful results for orbital implants have been reported^2^,^3^, complications, such as exposure, infection, migration, and extrusion, were observed in some cases^4^,^5^,^6^,^7^,^8^,^9^, which closely correlated with insufficient vascularization of the implant. For example, reports of orbital implant exposures were disclosed recently, with a rate ranging from 1.5 to 19.3%^10^. Vascularization of orbital implants is defined as the ingrowth of viable vascular connective tissue^11^, and the absence of a vascular network that is capable of distributing oxygen and other nutrients within the orbital implant is a major causal factor of complications.

The ability of biochemical and physical cues to promote the vascularization of a transplantable implant is a key issue in tissue engineering. A variety of approaches, including scaffold functionalization, material surface modification, and cell-based techniques, have been developed for this purpose^12^. For example, a fibrin scaffold loaded with vascular endothelial growth factor (VEGF) achieved more control over growth factor release and displayed increased vessel density^13^. Mihardja demonstrated that surface modification of the methylcellulose material with an RGD peptide could promote vascularization in a model of chronic ischaemic cardiomyopathy^14^. McGuigan assembled microscale modular components comprised of collagen gel rods seeded with endothelial cells (ECs) that enabled blood perfusion into the vascularized tissue^15^. Nonetheless, little attention has been paid to the vascularization of orbital implants to date.

The rapid vascularization of tissue-engineered grafts can be achieved by combining the strategies of stem cell therapy, material surface modification, and scaffold functionalization^16^. MSCs are outstanding candidates for use as seed cells due to their easy autologous acquisition, powerful *ex vivo* expansion, and long-term cryopreservation capacity. MSCs acquire several endothelial-like characteristics when cultured in EC growth supplement and stimulated by a matrix^17^,^18^. It is suggested that VEGF has a strong ability to induce the MSCs to differentiate into endothelial cells and promote vascularization, with an outcome that is better than that of the ECs^19^.

The layer-by-layer (LbL) self-assembly technique, which has the advantages of retaining the biomacromolecule activity and adaptation to substrates of variable sizes and shapes, has increasingly been used to immobilize biomacromolecules onto tissue-engineered scaffolds to support cell adhesion, survival, and differentiation^20^,^21^. In addition to synthetic polymers, many charged species, including proteins (enzymes, growth factors, and hormones), nanoparticles, and dye molecules, could easily be introduced into these multilayer films^22^,^23^,^24^. Collagen (COL)/heparin (HEP) multilayers provide an effective selection system for the surface modification of synthetic vascular grafts and show improved performance in clinical applications^25^. VEGF is one of the most well-studied growth factors, and it is a potent and specific angiogenic cytokine^26^. VEGF stimulates ECs by binding to a co-receptor, VEGFR-2 and to heparin/heparin sulfate, which induces matrix metalloproteinase (MMP) production, cell proliferation, and migration^27^,^28^. Grafts loaded with VEGF can be part of a useful strategy to stimulate and induce angiogenesis in tissue-engineered constructs^29^.

Here, we explore the advantages of using COL/HEP multilayers to load VEGF onto the HA scaffold surface to induce the differentiation of seeded MSCs to ECs and promote *in vivo* vascularization. The differentiated MSCs could be beneficial in the engineering of complex tissues, where the vascularization of the orbital implant is an essential feature. To clarify the influence of the VEGF-functionalized COL/HEP multilayers on vascularization *in vivo*, the experimental group was defined as the HA/(COL/HEP)_5_/VEGF/MSCs constructs, and the control groups were defined as the HA/VEGF/MSCs and HA constructs. The beneficial effects of the VEGF-functionalized COL/HEP multilayers were evaluated *in vitro* and *in vivo*.

## Results

### Preparation and characterization of the multilayer-assembled HA scaffolds

**Figure 1:**
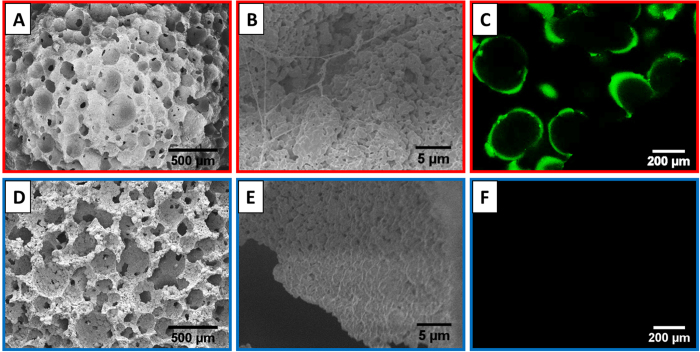
(**A,B,D,E**) SEM and (**C,F**) CLSM images of (**A–C**) the porous HA/(COL/HEP)_5_ scaffold and (**D–F**) the HA scaffold. In (**C**), the collagen used for the multilayer assembly was labelled with FITC for visualization.

Both the HA/(COL/HEP)_5_ scaffold and the HA scaffold had an open porosity of 75%, as determined by the mercury injection apparatus. SEM characterization showed that the average pore size of the HA scaffold decreased from 391.3 ± 92.6 μm to 316.8 ± 77.1 μm after being assembled with five COL/HEP multilayers. Many smaller pores of tens of microns in size were also visible on the walls of the large pores (Fig. 1). Filament-like structures appeared after assembly of the (COL/HEP)_5_ multilayers (Fig. 1B), which were consistently observed on the internal surface of the pore walls under CLSM (Fig. 1C, FITC-labelled collagen was used, green).

**Figure 2:**
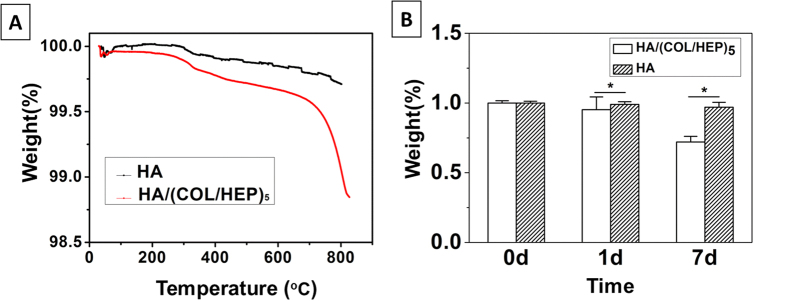
(**A**) Typical TGA curves of the HA/(COL/HEP)_5_ and HA scaffolds. (**B**) Mass loss of the HA/(COL/HEP)_5_ and HA scaffolds after immersion in PBS (pH 7.4) *in vitro*, as determined by TGA. The error bars represent the SD of the measurements performed on three samples. *p < 0.05.

Previous data showed that the thickness of five COL/HEP multilayers assembled on Si wafers is 35.5 ± 0.5 nm, as measured by spectroscopic ellipsometry^30^. Moreover, the typical layer density is approximately 1100 kg/m^3^ for most polyelectrolyte films using a quartz crystal microbalance with dissipation monitoring measurements^30^. However, these methods are not applicable to the 3D HA scaffold, and, thus, TGA was used to determine the assembled mass of the five COL/HEP multilayers. It was found to be 0.87% of the mass of the HA scaffold and COL/HEP multilayers (Fig. 2A). The inorganic HA was relatively stable, while the organic COL/HEP multilayers were thermo-degradable at 800 °C. Therefore, the excessive mass loss in the HA/(COL/HEP)_5_ group could only be attributed to the assembled multilayers. The COL/HEP multilayers were relatively stable, without significant mass loss after being incubated in phosphate-buffered saline (PBS) for 1 d (Fig. 2B). However, 7 d later, 43.7% of the COL/HEP multilayers were released into the medium, suggesting the partial destruction of the multilayers.

The amount of VEGF loaded into the scaffolds was measured using a rat VEGF 165 ELISA (anti-rat VEGF antibodies) kit, which could detect as little as 3.0 pg/mL of rat VEGF. The HA scaffold with a 6-mm diameter and a scaffold assembled with five COL/HEP multilayers was immersed in 200 μL of 1 μg/mL VEGF in PBS at 4 °C for 10 h, resulting in a VEGF loading dose of 3.39 ng/mm^3^ (191.40 ± 0.78 ng/scaffold). The loading dose of the uncoated HA scaffold was 0.16 ng/mm^3^ (9.31 ± 0.42 ng/scaffold).

### *In vitro* MSCs culture

The isolated cells were tested for the presence or absence of characteristic markers using flow cytometry. The MSCs expressed the typical antigens CD44 and CD29 (Fig. S1A, B), but they were negative for the typical lymphocytic marker CD45 (Fig. S1C) and the haematopoietic markers CD34 and CD11b (Fig. S1 D, E).

**Figure 3:**
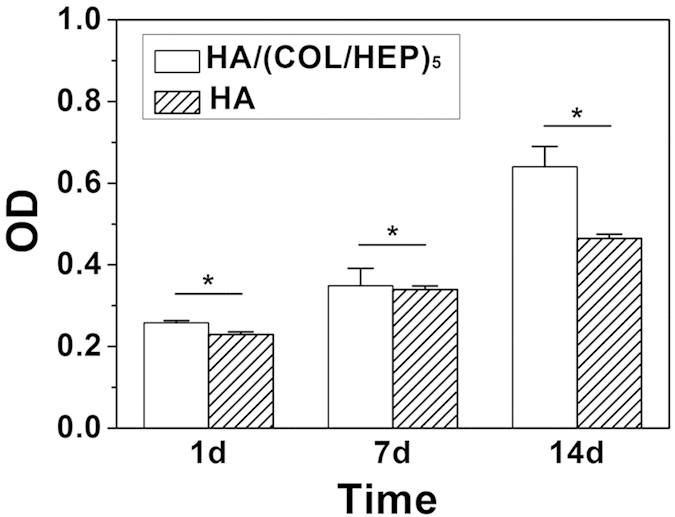
Cytoviability measured by the MTT assay. Both the HA/(COL/HEP)_5_ scaffold and the HA scaffold were seeded with MSCs at a density of 5 × 10^6^ cells/mL and cultured *in vitro*. The data are expressed as the means ± SD of triplicates. *p < 0.05.

The cytocompatibility of the HA/(COL/HEP)_5_ and HA scaffolds was evaluated by the MTT assay. Both types of scaffolds provided functional substrates for the adhesion and proliferation of MSCs. As shown in Fig. 3, the MSCs plated on all substrates exhibited increased cell proliferation over the 14-d culture period. In contrast, significantly higher viability was detected among the cells cultured on the HA/(COL/HEP)_5_ scaffold compared with those cultured on the HA scaffold at each time point.

**Figure 4:**
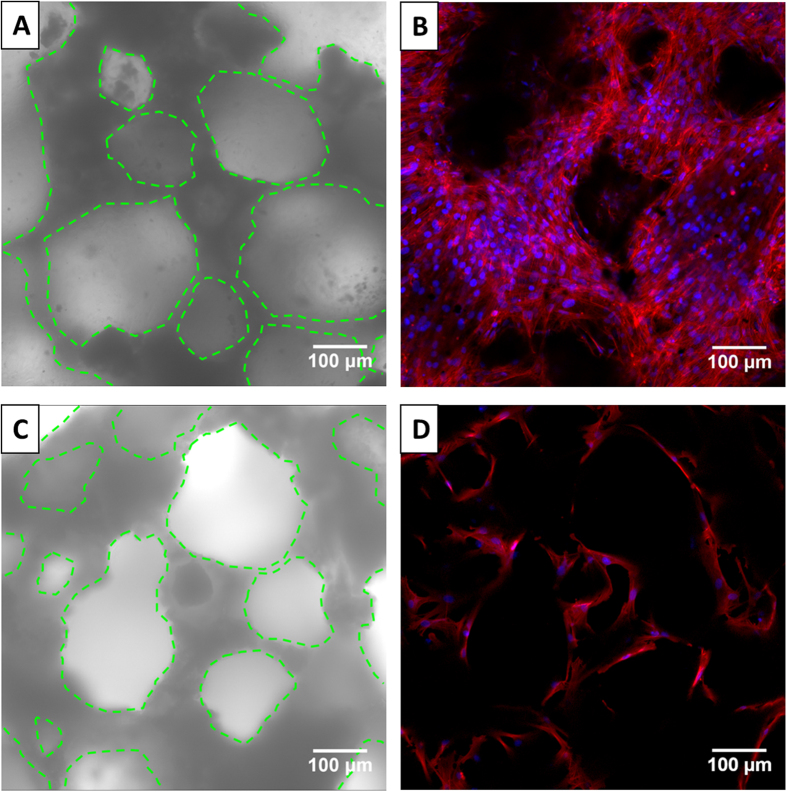
(**A,C**) Bright field and (**B,D**) CLSM images showing the distribution of the MSCs inside (**A,B**) the HA/(COL/HEP)_5_ scaffold and (**C,D**) the HA scaffold after 4 d of culture *in vitro*. The cell nuclei and actin were stained with DAPI (blue) and rhodamine phalloidin (red), respectively. The green dotted lines outline the contours of the pores.

After culturing for 4 d *in vitro*, the MSCs were distributed evenly throughout the entire space in the HA/(COL/HEP)_5_ scaffold (Fig. 4A,B), whereas the MSCs were observed only on the pore walls in the HA scaffold (Fig. 4C,D). More cells with a clustered morphology were observed inside the HA/(COL/HEP)_5_ scaffolds than inside the HA scaffolds, which is consistent with the results of the MTT assay.

**Figure 5:**
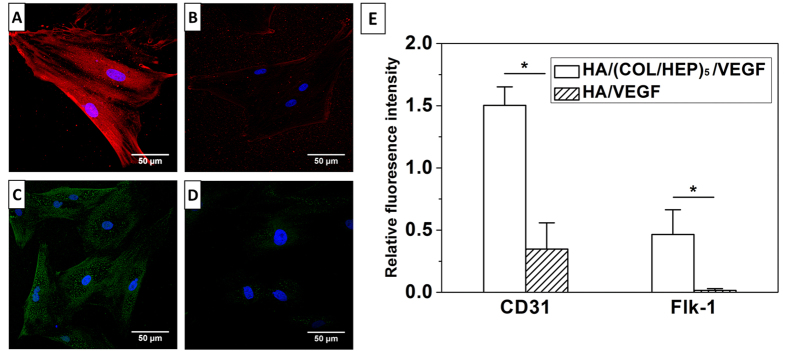
Immunofluorescence staining for the endothelial markers (**A,B**) CD31 (red) and (**C,D**) Flk-1 (green) in (**A,C**) MSCs cultured on the HA/(COL/HEP)_5_/VEGF and (**B,D**) HA/VEGF scaffolds. The cell nuclei were stained blue with DAPI. (**E**) Relative fluorescence intensity per cell. The data are expressed as the means ± SD of six samples. *p < 0.05.

To demonstrate that endothelial differentiation occurred, the expression of the CD31, Flk-1, and vWF mRNAs and proteins was evaluated quantitatively. After 7 d of culture *in vitro*, the HA/VEGF/MSCs group (Fig. 5B,D) showed very little specific staining for CD31 and Flk-1. In contrast, the overall fluorescence intensity of CD31 and Flk-1 in the HA/(COL/HEP)_5_/VEGF/MSCs group (Fig. 5A,C) was markedly enhanced. The relative fluorescence intensity per cell (Fig. 5E) confirmed the significant differences in CD31 and Flk-1 expression.

**Figure 6:**
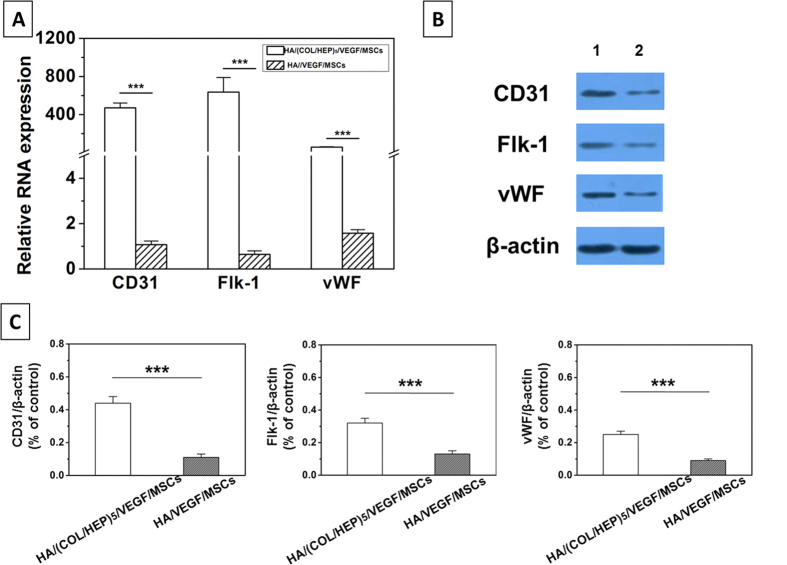
(**A**) qRT-PCR and (**B**) Western blot analyses of the expression levels of rat CD31, Flk-1, and vWF after the MSCs were cultured on (1) the HA/(COL/HEP)_5_/VEGF/MSCs and (2) HA/VEGF/MSCs constructs, respectively. The 18S rRNA was used as the internal control gene for the qRT-PCR analysis. (**C**) Semi-quantitative analysis of the CD31, Flk-1, and vWF levels from the western blot analysis (**B**). The data are expressed as the means ± SD of triplicates. ***p < 0.001.

To confirm the immunofluorescence data, qRT-PCR was performed. The expression levels of CD31, Flk-1, and vWF (Fig. 6A) were significantly upregulated in the HA/(COL/HEP)_5_/VEGF/MSCs group compared with those in the HA/VEGF/MSCs group (p < 0.001).

A western blotting analysis of the samples was also conducted to directly assess the levels of the CD31, Flk-1, and vWF proteins. As shown in Fig. 6B, the samples from the HA/(COL/HEP)_5_/VEGF/MSCs group showed darker bands compared with the corresponding bands of the HA/VEGF/MSCs group. A semi-quantitative analysis of the CD31, Flk-1, and vWF western blotting results confirmed that these factors were expressed at significantly higher levels in the HA/(COL/HEP)_5_/VEGF/MSCs group (Fig. 6C).

### *In vivo* implantation of the constructs

**Figure 7:**
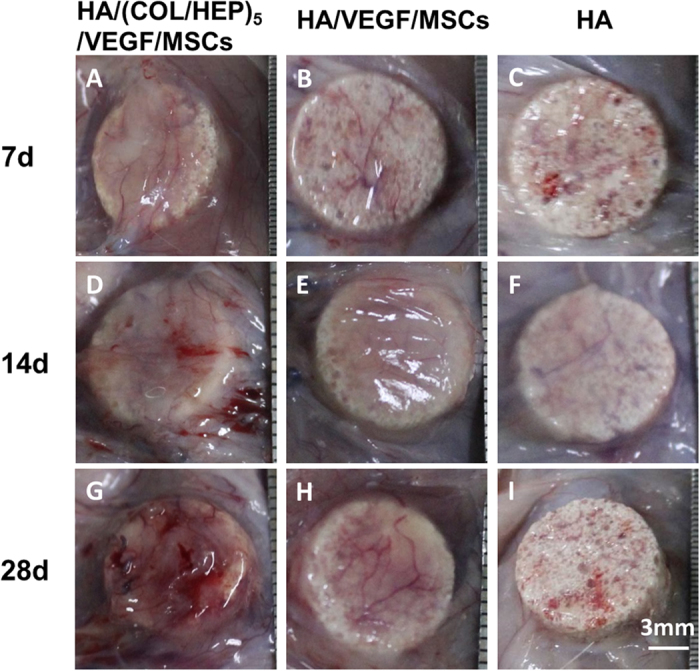
Gross observations of (**A,D,G**) the HA/(COL/HEP)_5_/VEGF/MSCs, (**B,E,H**) HA/VEGF/MSCs, and (**C,F,I**) HA implants after incubation *in vivo* for (**A–C**) 7, (**D–F**) 14, and (**G–I**) 28 d.

**Figure 8:**
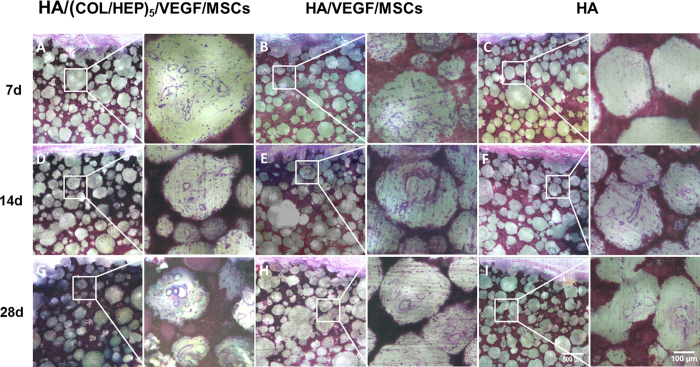
McNeal’s tetrachrome staining of sections of (**A,D,G**) the HA/(COL/HEP)_5_/VEGF/MSCs, (**B,E,H**) HA/VEGF/MSCs, and (**C,F,I**) HA implants after incubation *in vivo* for (**A–C**) 7, (**D–F**) 14, and (**G–I**) 28 d. The images on the right side of each row are higher magnification images of the selected regions marked by the square boxes.

**Figure 9:**
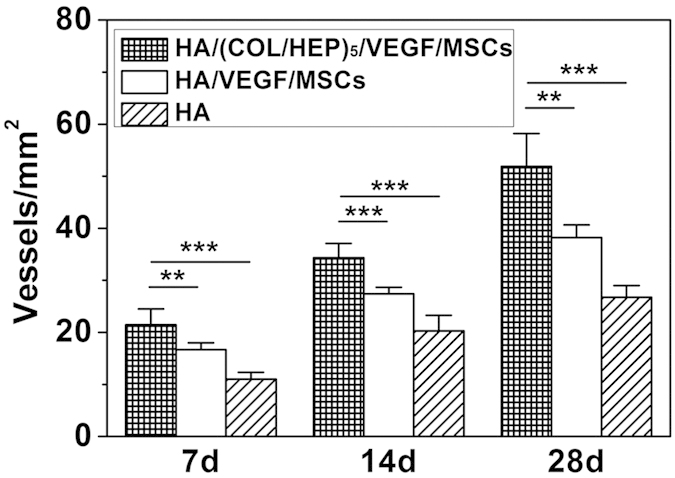
The number of newly formed blood vessels in tissue sections of the orbital HA/(COL/HEP)_5_/VEGF/MSCs, HA/VEGF/MSCs, and HA implants at different times (n ≥ 6). **p < 0.01; ***p < 0.001.

Having shown that the HA/(COL/HEP)_5_/VEGF scaffold could induce the MSCs to differentiate into endothelial cells *in vitro*, the HA/(COL/HEP)_5_/VEGF/MSCs, HA/VEGF/MSCs, and HA constructs were implanted beneath the panniculus carnosus muscle on the back of rats so that the vascularization around the implants could be observed (Fig. 7). After 7, 14, and 28 d of culture *in vivo*, the tissues around the constructs were carefully harvested to compare the vascular formation (Fig. 8). The vascularization of the constructs was revealed by representative McNeal’s tetrachrome staining and was quantified by assessing the new blood vessel density (Fig. 9). The number of newly formed blood vessels in the HA/(COL/HEP)_5_/VEGF/MSCs group was significantly higher than that in the HA and HA/VEGF/MSCs groups. The same results were obtained for cell proliferation and tube formation (Fig. 8). At all time points, the HA/(COL/HEP)_5_/VEGF/MSCs group had the highest vessel density, which was 21.4 ± 3.0/mm^2^, 34.4 ± 2.7/mm^2^, and 51.9 ± 6.3/mm^2^ on day 7, 14, and 28, respectively.

**Figure 10:**
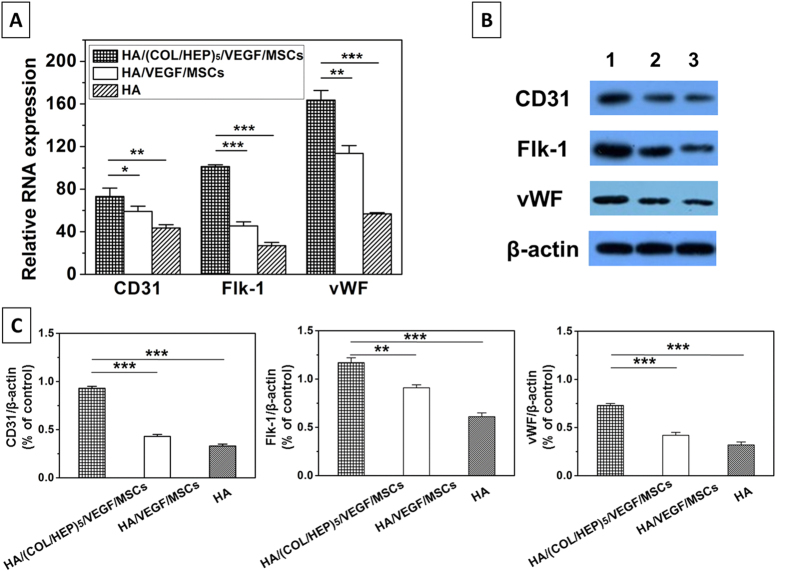
(**A**) qRT-PCR and (**B**) western blot analyses of the expression levels of rat CD31, Flk-1, and vWF in tissue sections of the orbital (1) HA/(COL/HEP)_5_/VEGF/MSCs, (2) HA/VEGF/MSCs, and (3) HA implants at 7 d. The 18S rRNA was used as the internal control gene for the qRT-PCR analysis. (**C**) Semi-quantitative analysis of the levels of the CD31, Flk-1, and vWF proteins from the western blot analysis (**B**). The data are expressed as the means ± SD of triplicates. *p < 0.05; **p < 0.01; ***p < 0.001.

To further confirm these results, qRT-PCR was used to quantify the *in vivo* expression of the rat CD31, Flk-1, and vWF mRNAs, which are known to be associated with angiogenesis. Figure 10A shows that CD31 expression was significantly upregulated in the HA/(COL/HEP)_5_/VEGF/MSCs group compared with the control groups (the HA/VEGF/MSCs and HA groups) at 7 d post-surgery. The expressions of Flk-1 and vWF (Fig. 10A) in the HA/(COL/HEP)_5_/VEGF/MSCs group showed a similar pattern to that of CD31; however, they were upregulated to a greater extent.

The western blot results (Fig. 10B) revealed similar expressions of the CD31, Flk-1, and vWF proteins. The bands from the HA/(COL/HEP)_5_/VEGF/MSCs group were more intense than the corresponding bands from the HA/VEGF/MSCs and HA groups at 7 d. A semi-quantitative analysis of the CD31, Flk-1, and vWF western blot results confirmed the significant differences, as shown in Fig. 10C.

## Discussion

A major limitation of the currently used orbital implants is slow vascularization, which may result in complications such as exposure. However, little attention has been given to enhancing the vascularization of orbital implant materials to date. Celik’s study indicated that coralline HA orbital implants show significantly faster and more homogenous vascularization than synthetic implants. Central vascularization patterns were seen in 62.5% of coralline orbital implants and 46.1% of the synthetic orbital implants^31^. In a recent *in vivo* study, the fibrovascular tissue ingrowth in the porous microstructure of the orbital implant showed an ability to significantly reduce the rate of exposure^32^. Tissue engineering represents a paradigm shift in medical treatment to overcome the disadvantages of the currently used orbital implants. In this study, a porous HA orbital scaffold coated with VEGF-functionalized COL/HEP multilayers was used as a 3D platform to improve angiogenesis, with VEGF as the stimulant and MSCs as the model cells.

To mimic the endothelial extracellular matrix (ECM), COL and HEP were used as the building blocks to cover the HA scaffolds with a multilayer thin film. Zhang demonstrated that assembled COL/HEP multilayers could improve endothelialization and haemocompatibility^33^. Although the assembly of COL and HEP multilayers on a 2D surface was reported previously^34^, a 3D porous scaffold coating has not been studied to date. Gong *et al*. used the LbL assembly technique to successfully deposit COL and chondroitin sulfate (CS) multilayers onto polylactide porous scaffolds to enhance the cell-material interaction^35^. In the present study, the SEM results (Fig. 1A,B,D,E), CLSM (Fig. 1C,F) images, and TGA results (Fig. 2A) demonstrated the successful assembly of the COL/HEP multilayers. Stability is very important when multilayer films are applied to porous scaffold coatings. The *in vitro* degradation rate of the multilayers within 7 d was 43.7% (Fig. 2B). Lin reported that the thickness of the (COL/HEP)_5_ multilayers on a 2D Si wafer surface decreased to approximately 85% of the initial thickness of the multilayers and then remained unchanged for 4 d^30^. In this study, the COL/HEP multilayers were successfully coated onto the 3D porous scaffold with a typical *in vitro* degradation rate.

The 3D multilayers serve as the substrate to support cell adhesion, survival, and differentiation. In the MTT assay (Fig. 3), the OD of the HA/(COL/HEP)_5_ scaffolds was higher than that of the unmodified scaffolds on days 1, 7, and 14. Both the MTT and CLSM (Fig. 4B,D) results indicated that the MSCs cultured on the HA/(COL/HEP)_5_ scaffolds exhibited much more proliferation than those on the HA scaffolds, eventually showing a significant improvement in the MSC density. The *in vitro* EC culture revealed that the COL/CS multilayer coating significantly improved cell attachment, proliferation, and viability^36^. Lanfer created aligned COL structures using a microfluidic set-up to guide the proliferation and differentiation of the resident MSCs^37^. The cell density is also a very important parameter for the endothelial differentiation of MSCs^38^. At higher cell densities, the cell-cell contact force and interactions, as well as the matrix elasticity, would be the main physical cues guiding MSC differentiation^39^. Li’s work suggests that a high local cell density has an effect on the differentiation status of MSCs, which, in turn, significantly affects the regeneration outcomes^40^.

The constituents of the matrix have an important impact on the reservoir and release behaviour of growth factors. The ELISA results showed that the VEGF loading doses of the coated HA/(COL/HEP)_5_ scaffold and uncoated HA scaffold were 3.39 ng/mm^3^ (191.40 ± 0.78 ng/scaffold) and 0.16 ng/mm^3^ (9.31 ± 0.42 ng/scaffold), respectively. The binding of VEGF to its two known receptors, KDR and Flt-1, is modulated by heparin or heparan sulfate^41^. The HEP-conjugated growth factor delivered by the COL-HEP scaffolds can enhance angiogenesis^27^,^42^. The VEGF-induced endothelial differentiation of MSCs is more effective in inducing the formation of new vessels. Therefore, we inferred that the binding of VEGF to the COL/HEP multilayers via the LbL assembled heparin would accelerate vascularization. However, it was reported that VEGF-loaded porous scaffolds increase the inflammatory response, which is likely attributed to an abnormal angiogenic response to the high local VEGF concentration^43^. In that study, the growth factor dose was rather high (300 ng VEGF/implant, 5.31 ng/mm^3^) compared with the 191.40 ± 0.78 ng/implant (3.39 ng/mm^3^) dose used in our study (150-mg implants were typically used in our study). Bigalke used a polylactide/1.0 μg VEGF-coated suture material, which enhanced cell viability *in vitro* and significantly increased vascularization^44^. These results demonstrated a dose-effect relationship of VEGF.

In this study, we compared the endothelial differentiation and vascular formation of two types of matrices, HA/(COL/HEP)_5_/VEGF and HA/VEGF scaffolds. After 7 d of induction *in vitro*, immunofluorescence staining (Fig. 5), qRT-PCR (Fig. 6A), and western blotting (Fig. 6B,C) demonstrated that the expression of the endothelial differentiation markers CD31, Flk-1, and vWF was markedly enhanced in the HA/(COL/HEP)_5_/VEGF/MSCs group. In the absence of the VEGF-functionalized COL/HEP multilayers, the HA/VEGF/MSCs group showed no statistically significant results regarding endothelial differentiation. Thus, we concluded that the interaction between VEGF and the COL/HEP multilayers resulted in the upregulation of the expression of the endothelial differentiation markers in MSCs. MSCs possess the capacity to differentiate into endothelial cells when they are cultured under high-ECM and endothelial differentiation conditions, and they display an EC-like phenotype^45^,^46^,^47^. When applied *in vivo*, these cells show greater angiogenic potential than native MSCs or ECs, which are usually used as the gold standard in vascularization studies^18^,^48^. Jay *et al*. provided the first comprehensive report that under appropriate *in vitro* environmental conditions, MSCs could be induced to undergo vasculogenic differentiation, culminating in microvessel formation^49^. Recent studies show that the *in vitro* scaffolds that are pre-cultured with human endothelial progenitors and MSCs promote the formation of vascular structures before implantation^50^,^51^.

To simulate the *in situ* environment of Tenon’s capsule and confirm the definite effects of the novel orbital implants, it was necessary to insert plate-shaped constructs beneath the panniculus carnosus muscle on the back of rats. From the macroscopic view, the HA/(COL/HEP)_5_/VEGF/MSC group showed better vascularization around the orbital implants. No exposure or inflammatory responses were observed in any of the three groups (Fig. 7). The *in vivo* qRT-PCR and western blot results confirmed that the HA/(COL/HEP)_5_/VEGF/MSC group also had the highest expression levels of the endothelial differentiation markers CD31, Flk-1, and vWF (Fig. 10). Although the general trends of vascularization were very similar *in vitro* and *in vivo*, the *in vivo* environment was much more complicated. Numerous extracellular proteins, growth factors, chemokines, cytokines, enzymes, and lipoproteins are involved in a variety of biological processes and can interact with HEP at the cell surface and in the ECM. HEP-protein interactions could regulate vascularization by binding to many extracellular proteins, growth factors, and receptors (e.g., α5β1 and αvβ3 integrins and VEGFR-2)^52^. Dai indicated that more VEGF could be immobilized on the HEP-modified demineralized bone matrices than could be bound naturally. The modified scaffolds have a greater biological activity in promoting cellular infiltration and capillary invasion in scaffolds *in vivo*^53^. Previous studies showed that VEGF only promotes transient vascularization and that the newly formed vascular network exhibits poor stability and may regress once the VEGF supply is exhausted^54^. However, HEP binds numerous growth factors and maintains their bioactivity for an extended period^55^. The HEP-modified scaffolds of mineralized COL exhibit favourable growth factor binding and release properties that are beneficial for stimulating vascularization^56^. In our study, compared with the HA/VEGF/MSCs (38.2 ± 2.4/mm^2^) and HA (26.7 ± 2.3/mm^2^) groups, the HA/(COL/HEP)_5_/VEGF/MSCs group (51.9 ± 6.3/mm^2^) exhibited a significant difference in the number of blood vessels, revealing that the endothelial differentiation of MSCs induced by the VEGF-functionalized multilayers had a positive effect on angiogenesis after day 28 (Fig. 9). In a previous *in vivo* study with a higher VEGF loading dose of 30.4 ng/mm^3^, a significant angiogenic capacity was found for VEGF-releasing polymeric scaffolds with a bioactive glass coating (53.79 vessels/mm^2^)^57^. For ceramic materials, the stimulation of angiogenesis by superficially adsorbed growth factors is temporally restricted to the first 2 weeks after biomaterial implantation. However, the long-term release of VEGF that is achieved with protein incorporation significantly enhanced angiogenesis between day 7 and 14, as well as between day 14 and 28, after biomaterial implantation^58^. In Chung’s study, HA orbital implants and HA-coated alumina orbital implants showed no significant difference in fibrovascular ingrowth^59^. Our *in vivo* experiments demonstrated that the HA scaffolds with MSCs and VEGF-functionalized COL/HEP multilayers had good vascularization. This design may be a key requirement for scaffolds that support vascular-engineered tissues.

## Conclusions

Here, HA/(COL/HEP)_5_/VEGF/MSCs scaffolds were designed and manufactured to promote vascularization *in vivo*, and their biological performance was evaluated in a rat model. The CLSM observations and cytocompatibility assays showed that the HA scaffolds covered with five COL/HEP multilayers supported the growth of a larger number of MSCs after 14 d of culture *in vitro*. The *in vitro* assays showed that the expression of the endothelial differentiation markers CD31, Flk-1, and vWF was significantly increased in the HA/(COL/HEP)_5_/VEGF/MSCs constructs (experimental) compared with the HA/VEGF/MSCs and HA/MSCs constructs (controls). After the constructs were implanted beneath the panniculus carnosus muscle on the back of the rats for 28 d, the experimental group exhibited a significantly larger number of newly formed blood vessels. Both the *in vitro* and *in vivo* assays showed that the VEGF-functionalized HA/(COL/HEP)_5_ scaffold induced the differentiation of MSCs into endothelial cells and promoted vascularization *in vivo*. Although additional longer-term analyses would lead to more confident conclusions, the biomembrane-mimic coating could represent a good foothold for the vascularization of orbital implants and thus has great potential for *in vivo* applications in the future.

## Methods

### Materials

The porous HA scaffolds were obtained from the Engineering Research Center in Biomaterials of Sichuan University, China. Collagen (COL, type I, from rat tail tendons, Shengyou Biotechnology Co., Ltd., China) was dissolved in acetic buffer (0.1 M HAc, 0.2 M NaCl) at 1 mg/mL. Heparin (HEP, porcine intestinal mucosa, Sigma, USA) was dissolved in a 0.2 M NaCl solution at 1 mg/mL. Dimethylthiazol diphenyltetrazolium bromide (MTT, Sigma, USA) was dissolved in PBS at 5 mg/mL. The polyelectrolyte solutions were filtered through 0.22-μm microfiltration membranes for sterilization. All other reagents were local products of analytical grade and were used as received.

### Multilayer fabrication

The porous HA scaffolds were used as substrates for multilayer assembly in the thermogravimetric analysis and in the *in vitro* and *in vivo* tests. Each experiment was initiated by COL adsorption for 20 min, followed by rinses with an acetic buffer solution, resulting in a stable, positively charged substrate surface^60^. The substrates were then incubated in the HEP solution for 20 min. The HEP-adsorbed substrates were then dipped into the COL solution for 20 min, followed by the same rinses. The alternating polyelectrolyte deposition circle was continued until five bilayers of COL/HEP were obtained.

The HA/(COL/HEP)_5_ and HA scaffolds were immersed in sterile PBS (pH 7.4) at 37 °C to test the *in vitro* degradation of the scaffolds. The PBS was changed every 2 d. At predetermined time intervals, five samples of each group were collected, rinsed with Millipore water, and freeze-dried. The mass of assembled multilayers and the weight loss of the multilayers *in vitro* were measured by thermogravimetric analysis (TGA, Perkin Elmer, TGA7, USA). The experiment was performed under an air atmosphere at a heating rate of 10 °C/min from 25 to 800 °C.

The porous structure of the scaffolds was characterized by scanning electron microscopy (SEM; JEOL JEM-200CX). The samples were sputter-coated with gold (Hitachi, E-1045) and observed under SEM. The pore size was measured by the ruler tool incorporated in the SEM software, and the average pore size was calculated from 50 pores. The porosity of the scaffolds was determined using a mercury porosimeter (Auto Pore, IV 9500). The total intrusion volume of mercury was recorded, and the porosity was analysed by taking into account the apparent volume of the scaffolds and the pore volume.

### Cytocompatibility

The cytocompatibility of the HA and HA/(COL/HEP)_5_ scaffolds was evaluated using the MTT assay. The scaffolds were sterilized in 75% ethanol solution for more than 4 h, and the ethanol solution was then exchanged with Millipore water. The sterilized scaffolds (6 mm in diameter and 2 mm in height) were placed into the wells of a 96-well plate, which exactly filled the diameter of each well and served as a 3D culture system for the MSCs. Forty microliters of the cell suspension (5 × 10^6^ cells/mL) were added into each well, and another 100 μL of culture medium was added after 4 h. The cells were cultured for up to 14 d with a change of medium every other day. On days 1, 7, and 14, the culture medium was removed and the cells were washed gently with PBS. New culture medium containing 20 μL of MTT (5 mg/mL) was added to each culture well and maintained for 4 h. The MTT was reduced to formazan crystals by the living cells. Finally, the culture medium was removed, and the cells were washed gently with PBS. After the samples were broken into small pieces by pounding in a mortar, dimethyl sulfoxide (DMSO) was added to extract the purple crystals. The absorbance at 570 nm was recorded with a microplate reader (Bio-Rad 680, USA).

### Preparation and characterization of the HA/(COL/HEP)_5_/VEGF/MSCs constructs

After the HA/(COL/HEP)_5_ scaffolds were incubated in 1 μg/mL VEGF (rat VEGF 165; PeproTech EC Ltd., UK) for 10 h, they were rinsed in PBS three times to remove all of the unbound growth factor. VEGF was applied to all scaffolds. The loading dose of VEGF was measured by a rat VEGF 165 ELISA Kit (Abcam, UK) according to the manufacturer’s procedures. The ELISA plate was read at 450 nm, and VEGF standards were used to create a calibration curve.

The MSCs were isolated from the bone marrow of young adult male Sprague-Dawley rats according to previously described procedures^61^, which were approved by the Committee on Animal Experimentation of Zhejiang University. Briefly, the bone marrow cells were obtained from rat femoral shafts by flushing them with 10 mL of culture medium (α-MEM medium supplemented with 10% bovine serum (GIBCO, USA), 100 U/mL penicillin, and 100 μg/mL streptomycin). The released cells were maintained and expanded for an additional passage in culture medium. After incubation at 37 °C and 5% CO_2_ for 5 d, the non-adherent cells were removed by washing twice with basal medium. The medium was then changed every 2 d until the cells reached approximately 90% confluence. The cells were retrieved by treating them with 0.25% trypsin and subcultured at a ratio of 1:3. The MSCs were used at passages 2 or 3. The detached MSCs were seeded onto the scaffold at a density of 4 × 10^7^ cells/mL.

The MSCs were characterized by flow cytometry using a panel of monoclonal antibodies. The cells were trypsinized, washed with PBS, and incubated with antibodies against CD44, CD29, CD34, CD45, and CD11b (all from Becton-Dickinson). The analysis was performed with a fluorescence-activated cell sorter (FACS) caliber flow cytometer (Becton Dickinson).

### Characterization of MSCs in each group

F-actin and the cell nuclei were stained to observe the distribution and morphology of the cells in each group by confocal laser scanning microscopy (CLSM, Zeiss, LSM 5 EXCITER, Germany). Briefly, after 4 d of culture *in vitro*, the scaffolds were washed three times with PBS and then fixed in 4% paraformaldehyde at 4 °C overnight. After three washes in PBS, the samples were immersed in 0.5% (v/v) Triton X-100/PBS for 10 min at 4 °C. After an additional three washes in PBS, the samples were incubated in 1% bovine serum albumin (BSA)/PBS for 80 min at 37 °C to block nonspecific interactions. Finally, the cells were stained with rhodamine phalloidin (Invitrogen) and 4′,6-diamidino-2-phenylindole (DAPI, Sigma) for 2 h at room temperature and then observed by CLSM.

### *In vitro* differentiation studies

The HA/(COL/HEP)_5_/VEGF/MSCs and HA/VEGF/MSCs constructs were incubated in α-MEM culture medium containing 10% FBS and were cultured at 37 °C with 5% CO_2_. The constructs were collected after 7 d and evaluated to determine the expression of the rat CD31, Flk-1, and von Willebrand factor (vWF) mRNAs and proteins using real-time quantitative polymerase chain reaction (qRT-PCR), immunofluorescence, and western blotting (see below), respectively.

For the immunofluorescence assay, the cells were fixed in 4% paraformaldehyde for 15 min and then blocked with 1% BSA for 20 min at room temperature. After an overnight incubation with the mouse anti-rat CD31 antibodies (Abcam, UK) and rabbit anti-rat Flk-1 (Abcam) antibodies at 4 °C, the cells were incubated with the appropriate secondary antibodies (AlexaFluor 594-conjugated goat anti-mouse or AlexaFluor 488-conjugated goat anti-rabbit; Invitrogen, USA) for 1 h at 37 °C. After washing with PBS, the samples were observed under CLSM. DAPI staining (blue) was applied to highlight the total nuclei. Six locations per scaffold were randomly photographed, and the relative fluorescence intensity per cell was determined using the ImageJ software.

### Animal experiments

**Figure 11:**
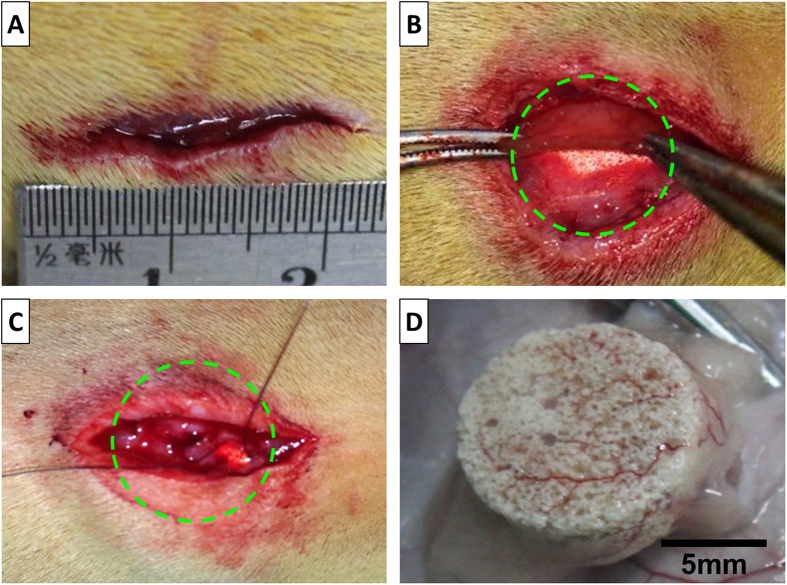
(**A**) A mid-sagittal incision was made on the dorsal surface of the Sprague-Dawley rats. (**B**) The implant construct (12 mm in diameter, 5 mm thick) was inserted beneath the panniculus carnosus muscle on the back of the rats. (**C**) The superficial fascia was closed with sutures. (**D**) The implant was harvested 28 d post-surgery to evaluate vascularization. The green dotted lines outline the area of the implant.

All of the animal experiments were approved by the Committee on Animal Experimentation of Zhejiang University. The methods were performed in accordance with the approved guidelines. Thirty male Sprague-Dawley rats (150–200 g, six weeks old) were used in this study. All of the rats were implanted with a construct from the three different groups: the HA/(COL/HEP)_5_/VEGF/MSCs (experimental) and HA/VEGF/MSCs and HA (controls) constructs. The rats were immobilized after the intraperitoneal administration of a sodium pentobarbital solution (w/v, 3%) at a dosage of 1 mL/kg. Under isoflurane anaesthesia, mid-sagittal incisions (Fig. 11A) were made on the dorsa of the rats. Each implant construct was inserted beneath the panniculus carnosus muscle on the back of the rats (Fig. 11B) so that the vascular formation could be observed around the implant. The superficial fascia (Fig. 11C) and the skin incisions were sutured closed. Three blocks were placed in each animal, and the implants were harvested at 7, 14, and 28 d (Fig. 11D). Digital images of the macroscopic views of the harvested implants were captured with a Canon D1200 camera.

### Histological analysis and quantification of blood vessels

The animals were sacrificed at 7, 14, and 28 d after implantation by an intraperitoneal injection of an overdose of sodium pentobarbital. The implants were immediately fixed in neutral buffered formalin, dehydrated with ascending concentrations of ethanol, and embedded in poly(methyl methacrylate). The embedded specimens were cut into 1000-μm-thick sections perpendicular to the bone using a saw microtome (Leica SP1600, Germany) and ground and polished to a final thickness of approximately 500 μm. Each specimen (five specimens from each group) yielded five to six serial sections. The slides were polished and stained using McNeal’s tetrachrome^62^. The mounted slides were examined under a light microscope (Nikon Eclipse 50i, Japan) at 40 × and 100 × magnification. The number of blood vessels in the vascular structures was counted in at least five different sections. Six locations per section were selected at random. All histological results were obtained by two independent researchers.

### qRT-PCR analysis

The *in vitro* and *in vivo* expression of the rat CD31, Flk-1, and vWF mRNAs in the HA/(COL/HEP)_5_/VEGF/MSCs, HA/VEGF/MSCs, and HA constructs was detected by qRT-PCR.

After culturing for 7 d, the samples were harvested, frozen in liquid nitrogen, homogenized, and treated with TRIzol (Invitrogen). The total RNAs from each sample were extracted using an RNeasy Mini Kit (Qiagen). Approximately 1 μg of the total RNAs was reverse-transcribed to produce complementary DNAs (cDNAs) using an Omniscript RT Kit (Qiagen). qRT-PCR was performed as previously described^63^, and 18S rRNA was used as the internal control gene. The primer sequences for the rat CD31 gene were as follows: forward (5′−3′) CAGCGTTCAACAGAGCCAGCAT (product size 80 bp) and reverse (5′−3′) CTTCCACGGAGCAAGAAAGACT. The primer sequences for the rat Flk-1 gene were as follows: forward (5′−3′) GTCAAGTGGCGACGGTAAAGG (product size 78 bp) and reverse (5′−3′) GTGTGGCAAGACAGAAGTGGAGTT. The primer sequences for the rat vWF gene were as follows: forward (5′−3′) GTCGGAAGAGGAAGTGGACATT (product size 135 bp) and reverse (5′−3′) GGGCACACGCATGCGCTCTGTA. The primer sequences for the rat 18S gene were as follows: forward (5′−3′) GAATTCCCAGTAAGTGCGGGTCATA (product size 105 bp) and reverse (5′−3′) CGAGGGCCTCACTAAACCATC. An ABI 7300 real-time PCR system (Applied Biosystems) with SYBR Green PCR master mix (Applied Biosystems) was used to perform the qRT-PCR, and the conditions were 45 cycles for 10 s at 95 °C and 25 s at 62 °C. Three samples in each group were analysed at each time point.

### Western blot assay

The *in vitro* and *in vivo* expression of the rat CD31, Flk-1, and vWF proteins was assessed by western blotting. Seven days after implantation, the samples were harvested and stored in liquid nitrogen until evaluation. The frozen samples were completely homogenized in RIPA lysis buffer (50 mM Tris-HCl, 0.1% Triton X-100, 2 mM ethylenediaminetetraacetic acid (EDTA), 100 mM NaCl, and 1 mM protease inhibitors) for 30 min at 4 °C and separated by sodium dodecyl sulfate-polyacrylamide gel electrophoresis (SDS-PAGE).

After being transferred to a poly(vinylidene fluoride) membrane (Millipore, MA, USA), the membranes were incubated with a solution of 5% milk powder in Tris-buffered saline (TBS) to prevent nonspecific interactions. Then, the membranes were incubated overnight with the antibodies and detected using an ECL (ECL western blotting substrate, Pierce, USA) system. The specific primary antibodies used for this experiment were mouse anti-rat CD31 (Abcam, UK), rabbit anti-rat Flk-1 (Abcam, UK), and mouse anti-rat vWF (Santa Cruz, USA) antibodies.

### Statistical analysis

The data are expressed as the means ± SD. The statistical significance between groups was determined by the two-population Student’s t test using the Origin software. The statistical significance was set at p < 0.05.

## Additional Information

**How to cite this article**: Jin, K. *et al*. *In vivo* vascularization of MSC-loaded porous hydroxyapatite constructs coated with VEGF-functionalized collagen/heparin multilayers. *Sci. Rep.* **6**, 19871; doi: 10.1038/srep19871 (2016).

## Supplementary Material

Supplementary Information
