# Supplementary material for: In vivo vascularization of MSC-loaded porous hydroxyapatite constructs coated with VEGF-functionalized collagen/heparin multilayers
Source: Sci Rep. 2016 Jan 22;6:19871. doi: 10.1038/srep19871 (PMC4726420; doi:10.1038/srep19871)
Supplement: Supplementary Information [file srep19871-s1.pdf]

Supplementary information

In vivo vascularization of MSCs-loaded porous hydroxyapatite constructs coated with VEGF-functionalized collagen/heparin multilayers

Kai Jin,[a] Bo Li,[b] Lixia Lou,[a] Yufeng Xu,[a] Xin Ye,[a] Ke Yao,[a] Changyou Gao,[b*] Juan Ye,[a**]

[a] *Department of Ophthalmology, the Second Affiliated Hospital of Zhejiang University, College of Medicine, Hangzhou 310009, China.*

[b] *MOE Key Laboratory of Macromolecular Synthesis and Functionalization, Department of Polymer Science and Engineering, Zhejiang University, Hangzhou 310027, China.*

Figure S1

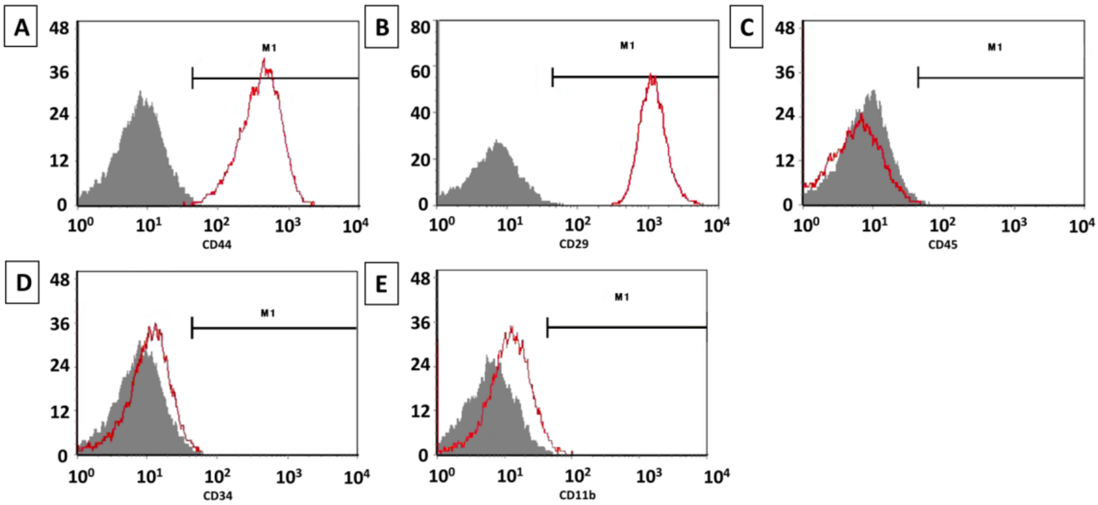

Fig S1. The presence or absence of characteristic markers on MSCs, as tested by fluorescence-activated cell sorting (FACS) analysis. (A) CD44, (B) CD29, (C) CD34, (D) CD45 and (E) CD11b.
